# Supplementary material for: Influence of Sex on Urinary Organic Acids: A Cross-Sectional Study in Children
Source: Int J Mol Sci. 2020 Jan 16;21(2):582. doi: 10.3390/ijms21020582 (PMC7013514; doi:10.3390/ijms21020582)
Supplement: Supplementary file 1 [file ijms-21-00582-s001.pdf]

Table S1. Metabolites detected in less than 20% of the male and female cohorts.

Metabolites	Age Group 1 (1–6 Months)		Age-Group 2 (7–12 Months)		Age-Group 3 (13–24 Months)		Age-Group 4 (25–36 Months)		% M-F
	Males (N = 37)	Females (N = 37)	Males (N = 30)	Females (N = 28)	Males (N = 43)	Females (N = 38)	Males (N = 42)	Females (N = 36)	
2-hydroxy-sebacate	0 (5)	0 (1)	0 (1)	0 (4)	0 (3)	0 (1)	0 (3)	0 (0)	7.9-4.3
2-keto-3-methylvalerate	0 (2)	0 (4)	0 (1)	0 (6)	0 (4)	0 (4)	0 (3)	0 (2)	6.6-11.5
2-keto-isovalerate	0 (1)	0 (1)	0 (0)	0 (1)	0 (0)	0 (0)	0 (0)	0 (1)	0.66-2.1
2-methylbutyrylglycine	0 (0)	0 (3)	0 (0)	0 (0)	0 (1)	0 (1)	0 (3)	0 (9)	2.6-9.3
3-hydroxy-3methyl-glutarate	0 (2)	0 (0)	0 (0)	0 (2)	0 (2)	0 (1)	0 (0)	0 (0)	2.6-3.6
3-hydroxy-dodecanoate	0 (0)	0 (0)	0 (0)	0 (2)	0 (0)	0 (1)	0 (0)	0 (2)	0-3.6
3-hydroxy-butyrate	0 (4)	0 (7)	0 (7)	0 (8)	0 (8)	0 (5)	0 (6)	0 (3)	16.4-16.5
3-hydroxy-valerate	0 (1)	0 (1)	0 (0)	0 (0)	0 (0)	0 (0)	0 (0)	0 (0)	0.7-0.4
3-methylcrotonylglycine	0 (0)	0 (3)	0 (0)	0 (0)	0 (0)	0 (0)	0 (1)	0 (0)	0.7-2.1
4-hydroxy-butyrate	0 (0)	0 (0)	0 (1)	0 (0)	0 (0)	0 (0)	0 (0)	0 (0)	0.7-0
4-hydroxy-phenylpyruvate	0 (8)	0 (4)	0 (2)	0 (5)	0 (6)	0 (7)	0 (4)	0 (9)	13.1-17.9
5-hydroxy-indoleacetate	0 (1)	0 (2)	0 (0)	0 (0)	0 (0)	0 (0)	0 (0)	0 (0)	0.7-1.4
7-hydroxy-octanoate	0 (1)	0 (1)	0 (0)	0 (1)	0 (0)	0 (1)	0 (1)	0 (2)	1.3-3.6
Homogentisate	0 (1)	0 (0)	0 (0)	0 (0)	0 (0)	0 (0)	0 (0)	0 (0)	0.7-0
Homovanillate	0 (9)	0 (3)	0 (3)	0 (3)	0 (2)	0 (4)	0 (5)	0 (3)	12.5-9.3
Isovalerylglycine	0 (0)	0 (1)	0 (1)	0 (2)	0 (0)	0 (3)	0 (1)	0 (11)	1.3-12.3
Isobutyrylglycine	0 (2)	0 (4)	0 (2)	0 (2)	0 (9)	0 (3)	0 (5)	0 (8)	11.8-12.2
Malonate	0 (3)	0 (7)	0 (2)	0 (2)	0 (0)	0 (2)	0 (2)	0 (2)	4.6-9.3
Methylcitrate	0 (0)	0 (0)	0 (0)	0 (0)	0 (0)	0 (1)	0 (0)	0 (1)	0-1.4
N-acetyl-tyrosine	0 (6)	0 (1)	0 (2)	0 (2)	0 (1)	0 (2)	0 (2)	0 (1)	7.2-4.3
Phenylacetate	0 (0)	0 (0)	0 (0)	0 (0)	0 (0)	0 (1)	0 (0)	0 (1)	0-1.4
Phenylpyruvate	0 (0)	0 (0)	0 (0)	0 (0)	0 (0)	0 (1)	0 (0)	0 (0)	0.7-0.7
Vanilmandelate	0 (7)	0 (8)	0 (6)	0 (6)	0 (4)	0 (5)	0 (2)	0 (2)	12.5-15.1
3,4-dihydroxy-butyrate	0 (0)	0 (0)	0 (1)	1 (14)	0 (0)	0 (18)	0 (0)	0 (15)	0.7-33.8
5-hydroxy-caproate	0 (6)	0 (11)	0 (7)	0 (13)	0 (11)	0 (5)	0 (3)	0 (8)	17.7-26.6

Values (mmol/mol creatinine) are expressed as median value; in round brackets the number of subject in which the metabolite was detected; %M-F represents the percentage calculated on the total of male and female populations, respectively.

Table S2. Intra-sex analysis of male cohorts of different class of age. Values (median values and IQR) are expressed as mmol/mol creatinine.

Metabolites	Age-Group 1 (1–6 Months) (N = 37)	Age-Group 2 (7–12 Months) (N = 30)	Age-Group 3 (13–24 Months) (N = 43)	Age-Group 4 (25–36 Months) (N = 42)	P Value (Kruskall-Wallis Test)	Multiple Comparisons (Dunn's Test)
2-ethyl-3-hydroxy-propionate	0.0 (0-0.4)	0.0 (0-0.9)	0.0 (0-3.5)	0.0 (0-2.8)	0.022	None
2-hydroxy-glutarate	2.6 (1.0-4.0)	2.1 (1.0-3.7)	2 (1.7-2.9)	1.2 (1.0-2.0)	NS	
2-hydroxy-isobutyrate	0.0 (0-3.0)	0.0 (0.0-6.0)	2.8 (0-7.8)	0.0 (0-6.8)	NS	
2-keto-isocaproate	0.0 (0-0.2)	0.0 (0.0-0.0)	0.0 (0-0.6)	0.0 (0-0)	NS	
2methyl-3-hydroxy-butyrate	0.0 (0-0.8)	0.0 (0-1.2)	1.0 (0.0-3.0)	0.0 (0-2.3)	NS	
3-hydroxy-adipate	0.0 (0-1.0)	0.0 (0.0-2.0)	0.0 (0-25.0)	0.0 (0-1.0)	NS	
3-hydroxy-isobutyrate	14.0 (8.1-18.0)	13.0 (10.0-17.0)	14.0 (10.7-18.1)	13.7 (11.0-18.1)	NS	
3-hydroxy-isovalerate	0.0 (0.0 -0.0)	0.0 (0.0 -1.3)	0.0 (0.0-2.2)	0.0 (0.0-2.7)	NS	
3-hydroxy-propionate	3.3 (2.0-4.0)	3.0 (2.1-4.0)	3.0 (2.0-4.0)	3 (1.8-4.9)	NS	
3-hydroxy-sebacate	0.0 (0.0-0.0)	0.0 (0.0-0.6)	0.0 (0-0.6)	0.0 (0.0-0.0)	NS	
3-methyl-glutaconate	0.0 (0.0-4.0)	2 (0.0-6.0)	0.0 (0.0-3.0)	2.0 (0.0-4.0)	NS	
3-methyl-glutarate	0.0 (0.0-1.0)	1.0 (0.0-1.5)	0.0 (0.0-2.0)	0.0 (0.0-1.0)	NS	
4-hydroxy-phenylacetate	25 (14.01-41.6)	22.6 (16.0-34.0)	22.4 (15.8-32.0)	21.8 (15.0-32.9)	NS	
4-hydroxy-phenyllactate	1.0 (0.0-2.0)	0.4 (0.0-1.0)	0.5 (0.0-1.5)	0.1 (0.0-1.0)	NS	
Adipate	11.0 (6.9-15.8)	8.9 (6.5-13.0)	9.8 (7.0-13.8)	7.6 (5.0-10.0)	NS	
Alpha-ketoglutarate	17.5 (4.30-36.3)	10.2 (0-25.3)	14.75 (0-35.7)	7.25 (0-19.5)	NS	
Azelate	0.0 (0-3.8)	1.6 (0-4.4)	2.2 (0-3.9)	1.0 (0-3.2)	NS	
Cis-Aconitate	18.0 (7.0-30.6)	16.0 (8.0-26.0)	18.9 (7.0-34.9)	14.9 (7.0-21.5)	NS	
Citrate	0.0 (0.0-22.7)	3.0 (0.0-21.0)	(0.0-5.7)	0.0 (0.0-7.2)	NS	
Ethylmalonate	4.0 (2.0-7.1)	4.8 (2.0-7.0)	3.0 (2.0-4.1)	3.0 (2.0-6.0)	NS	
Fumarate	3 (0.15-5.69)	1.52 (0.0-2.0)	1.70 (0.12-2.5)	0.6 (0.0-2.0)	<0.001	1 vs 4
Glutarate	3.5 (1.7-4.9)	3 (2.0-5.0)	3.6 (2.9-5.9)	2.8 (1.9-4.0)	NS	
Glycerate	0.0 (0.0-0.0)	0.0 (0.0-0.9)	0.0 (0.0-26.0)	0.0 (0.0-0.0)	NS	

Glycolate	18.0 (10.0-24.7)	20.3 (13.0-27.4)	21.6 (12.3-30.5)	20.1 (12.8-33.8)	NS	
Hippurate	24.3 (12.5-56.2)	33.8 (19.0-63.0)	79.9 (45.1-173.8)	87.7 (51.0-163.0)	<0.001	1 vs 4<br>2 vs 4<br>1 vs 3<br>2 vs 3
Lactate	28.0 (19.2-35.5)	25.0 (16.4-32.9)	19.6 (17.8-27.2)	21.2 (18.7-24.8)	NS	
Malate	0.0 (0.0-0.7)	0.0 (0.0-1.1)	0.0 (0.0-1.2)	0.0 (0.0-0.0)	NS	
Methylmalonate	1.0 (0-4.3)	0.9 (0-3.1)	0.0 (0.0-2.3)	0.0 (0.0-1.6)	NS	
Methylsuccinate	0.0 (0.0-1.9)	0.0 (0.0-3.0)	2.7 (0-4.2)	0.0 (0-2.9)	NS	
Oxalate	0.0 (0.0-3.5)	0.5 (0-2.8)	0.0 (0.0-0.0)	0.0 (0.0-0.0)	0.002	None
Palmitate	6.0 (4.0-9.3)	5.1 (3.0-8.7)	5.0 (4.0-8.6)	5.0 (3.0-7.5)	NS	
Pimelate	0.0 (0.0-2.0)	0.0 (0.0-1.3)	0.0 (0.0-2.0)	0.0 (0.0-1.5)	NS	
Pyroglutamate	14.4 (6.0-22.2)	14.51 (8.6-22.0)	13.9 (9.0-18.9)	11.4 (5.0-15.0)	NS	
Pyruvate	25.0 (16.9-30.6)	19.0 (14.0-28.0)	25.1 (18.0-31.1)	22.5 (15.6-27.6)	NS	
Sebacate	0.0 (0.0-1.0)	0 (0.0-0.8)	0.0 (0.0-0.0)	0.0 (0.0-0.0)	NS	
Stearate	0.0 (0.0-6.5)	0.0 (0.0-4.1)	0.0 (0.0-8.1)	0.0 (0.0-5.6)	NS	
Suberate	6.0 (2.9-11.5)	3.0 (1.8-5.0)	2.0 (1.1-4.9)	2.0 (0.9-3.1)	<0.001	1 vs 3<br>1 vs 4
Succinate	58.4 (15.7-69.5)	34.0 (22.0-42.0)	29.0 (19.8-38.0)	19.0 (14.0-27.0)	<0.001	1 vs 4<br>2 vs 4
Tiglylglycine	0.0 (0.0-0.0)	0.0 (0.0-0.0)	0.0 (0.0-0.8)	0.0 (0.0-0.4)	0.017	None
Uracil	0.0 (0.0-0.0)	0.0 (0.0-0.8)	0.0 (0.0-2.3)	0.0 (0.0-0.5)	0.033	None

Table S3. Intra-sex analysis of female cohorts of different classes of age. Values (median values and IQR) are expressed as mmol/mol creatinine.

**Metabolites**	**Age-Group 1 (1–6 Months) (N = 37)**	**Age-Group 2 (7–12 Months) (N = 28)**	**Age-Group 3 (13–24 Months) (N = 38)**	**Age-Group 4 (25–36 Months) (N = 36)**	**P Value (Kruskall-Wallis Test)**	**Multiple Comparisons (Dunn's Test)**
2-ethyl-3-hydroxy-propionate	1.0 (0.0-2.2)	1.01 (0.0-4.4)	0.0 (0.0-0.0)	0.0 (0.0-4.5)	0.006	2 vs 3
2-hydroxy-glutarate	1.0 (0.1-2)	2.5 (1.2-5.1)	2.8 (0.0-4.9)	2.0 (1.0-3.4)	0.004	1 vs 2<br>1 vs 3
2-hydroxy-isobutyrate	4.0 (0.0-6.1)	5.5 (0.0-9.3)	0.0 (0.0-9.6)	0.0 (0-9.0)	ns	
2-keto-isocaproate	0.0 (0.0-0.1)	0.0 (0.0-0.7)	0.0 (0.0-0.0)	0.0 (0.0-0.6)	ns	
2methyl-3-hydroxy-butyrate	1.0 (0-1.8)	1.9 (0.0-4.1)	0.0 (0.0-3.0)	0.0 (0.0-3.9)	ns	
3-hydroxy-adipate	0.0 (0-0.9)	0.0 (0.0-4.8)	0.0 (0.0-1.1)	0.0 (0.0-0.7)	ns	
3-hydroxy-isobutyrate	9.0 (6.9-13.4)	13.7 (10.0-15.9)	14.6 (7.1-20.5)	16.0 (12.0-22.5)	<0.001	2 vs 4
3-hydroxy-isovalerate	0.0 (0.0-1.3)	0.0 (0.0-3.2)	0.0 (0.0-0.0)	2.0 (0.0-4.0)	0.04	none
3-hydroxy-propionate	2.0 (1.5-4.0)	2.9 (2.0-4.0)	3.8 (2.4-6.3)	4.2 (3.0-5.0)	0.003	2 vs 4
3-hydroxy-sebacate	0.0 (0.0-0.0)	0.0 (0.0-0.4)	0.0 (0.0-0.0)	0.0 (0.0-0.0)	0.024	none
3-methyl-glutaconate	0.0 (0.0-0.2)	3.5 (1.8-7.6)	6.2 (2.0-9.0)	3.5 (1.9-7.0)	<0.001	1 vs 2<br>1 vs 3<br>1 vs 4
3-methyl-glutarate	0.8 (0-1.3)	0.2 (0.0-1.1)	0.0 (0.0-1.7)	0.0 (0.0-0.9)	ns	
4-hydroxy-phenylacetate	16.0 (11.0-24.1)	34.2 (15.5-59.1)	26.0 (10.4-35.8)	26.5 (13.4-47.4)	0.014	1 vs 2
4-hydroxy-phenyllactate	0.5 (0.0-1.0)	1.1 (0.0-2.3)	1.0 (0.0-2.0)	0.7 (0.0-1.7)	ns	
Adipate	7.1 (5.0-11.6)	10.3 (5.3-16.1)	8.2 (4.9-11.0)	8.7 (6.0-10.7)	ns	
Alpha-ketoglutarate	18.0 (7.5-28.9)	30.0 (14.8-64.7)	27.0 (12.0-58.0)	17.7 (5.7-39.5)	0.015	1 vs 2
Azelaate	1.2 (0-2.1)	3.6 (1.0-6.3)	0.0 (0.0-4.0)	0.0 (0.0-1.95)	<0.001	2 vs 3<br>2 vs 4
Cis-Aconitate	8.3 (2.0-23.6)	24.2 (8.0-40.7)	16.6 (8.0-28.8)	14.0 (3.0-28.3)	0.026	1 vs 2
Citrate	0.0 (0.0-4.1)	3.9 (0.0-33.7)	4.5 (0.0-20.0)	0.0 (0.0-0.9)	0.001	2 vs 4<br>3 vs 4
Ethylmalonate	2.4 (1.6-3.8)	4.33 (2.5-6.3)	4.00 (2.5-7.0)	3.0 (2.0-5.0)	0.006	1 vs 2<br>1 vs 3
Fumarate	1.0 (0.0-1.5)	2.4 (1.2-4.3)	1.2 (0.0-2.6)	1.6 (0.0-2.9)	0.003	1 vs 2
Glutarate	2.0 (1.0-3.0)	3.5 (2.0-5.7)	3.0 (1.9-5.0)	3.4 (1.5-4.8)	0.002	1 vs 2<br>1 vs3<br>1 vs 4
Glycerate	0.0 (0.0-0.0)	0.0 (0.0-2.0)	0.0 (0-1.1)	0.0 (0.0-0.0)	ns	
Glycolate	21.2 (10.5-29.0)	20.2 (14.2-33.9)	26.9 (18.0-46.1)	26.0 (15.5-34.7)	ns	
Hippurate	75.0 (31.7-157.4)	57.3 (13.5-131.5)	40.6 (14.0-103.0)	62.6 (31.5-121.5)	ns	
Lactate	18.84 (13.79-22.30)	28.02 (22.80-35.82)	24.84 (16.21-38.44)	20.4 (14.0-26.0)	<0.001	1 vs 2<br>2 vs 4

Malate	0.0 (0-0.1)	0.0 (0-1.8)	0.0 (0.0-0.0)	0.0 (0-0.6)	ns	
Methylmalonate	0.8 (0.0-1.7)	0.0 (0.0-1.9)	0.0 (0.0-0.0)	0.0 (0-1.3)	ns	
Methylsuccinate	2.4 (0-3.1)	4.4 (0.0-5.3)	2.3 (0.0-5.7)	0.0 (0.0-4.6)	ns	
Oxalate	0.0 (0.0-0.8)	0.0 (0.0-6.4)	0.0 (0.0-6.8)	0.0 (0.0-0.5)	ns	
Palmitate	5.0 (3.5-7.2)	7.3 (4.0-10.5)	6.6 (3.0-10.8)	8.1 (3.0-18.2)	ns	
Pimelate	1 (0-1.8)	2.2 (0-3.6)	0.0 (0.0-1.9)	0.0 (0.0-1.6)	0.007	2 vs 4
Pyroglutamate	9.0 (5.0-12.3)	20.1 (6.7-27.1)	13.8 (10.0-21.4)	14.0 (6.6-17.1)	0.007	1 vs 2<br>1 vs 3
Pyruvate	18.0 (14.0-23.6)	29.1 (17.3-35.1)	30.3 (12.1-40.0)	24.9 (19.0-30.7)	0.025	1 vs 2
Sebacate	0.0 (0.0-0.0)	0.0 (0-1.8)	0.0 (0.0-0.0)	0.0 (0.0-0.0)	<0.001	1 vs 2<br>2 vs 3
Stearate	4.44 (0-6.61)	6.31 (0-9.75)	0.0 (0.0-9.0)	0.0 (0.0-12.1)	ns	
Suberate	1.7 (0.6-3.0)	3.1 (0.6-6.2)	2.0 (1.0-3.0)	2.3 (1.0-3.0)	ns	
Succinate	17.0 (11.9-24.1)	28.5 (20.2-49.6)	23.3 (15.0-33.0)	20.2 (14.0-34.4)	ns	
Tiglylglycine	0.0 (0.0-0.1)	0.0 (0.0-0.0)	0.0 (0.0-0.0)	0.0 (0.0-0.9)	ns	
Uracil	0.0 (0.0-0.9)	0.0 (0.0-2.8)	0.0 (0-3.3)	0.0 (0.0-2.4)	ns	

Table S4. Metabolic pathway analysis which includes the tested urinary metabolites in male individuals.

Pathway Name	Match Status	p	-log(p)	Holm p	FDR	Impact	Metabolites
Phenylalanine metabolism	3(45)	0.0001	9.0	0.0094014	0.0094014	0.0315	Hippurate; Succinate; Fumarate
Citrate cycle (TCA cycle)	2(20)	0.0010	6.9	0.076216	0.037206	0.0313	Succinate; Fumarate
Alanine, aspartate and glutamate metabolism	2(24)	0.0014	6.5	0.10883	0.037206	0.00285	Succinate; Fumarate
Butanoate metabolism	2(40)	0.0039	5.5	0.29824	0.077465	0.03545	Succinate; Fumarate
Glyoxylate and dicarboxylate metabolism	2(50)	0.0060	5.1	0.45717	0.096246	0.12894	Succinate; Oxalate

Table S5. Metabolic pathway analysis which includes the tested urinary metabolites in female individuals.

Pathway Name	Match Status	p	-log(p)	Holm p	FDR	Impact	Metabolites
Citrate cycle (TCA cycle)	5(20)	0.0000	17.4	0.022834	0.022834	0.31123	Alpha-ketoglutarate; Cis-aconitate; Citrate; Pyruvate; Fumarate
Glyoxylate and dicarboxylate metabolism	4(50)	0.0001	9.2	0.0081165	0.0041096	0.00643	Cis-aconitate; Alpha-ketoglutarate; Citrate; Pyruvate
Alanine. aspartate and glutamate metabolism	3(24)	0.0002	8.4	0.018214	0.006227	0.00285	Pyruvate; Alpha-ketoglutarate; Fumarate
Butanoate metabolism	3(40)	0.0011	6.8	0.083475	0.021682	0.10287	Pyruvate; Alpha-ketoglutarate; Fumarate

Table S6. Age group I: significant metabolic pathways.

Pathway Name	Match Status	p	-log(p)	Holm p	FDR	Impact	Metabolites
Phenylalanine metabolism	3(45)	0.0019	6.2	0.15403	0.15403	0.0315	Hippurate; Succinate; Fumarate
Citrate cycle (TCA cycle)	2(20)	0.0056	5.2	0.44428	0.17139	0.0313	Succinate; Fumarate
Alanine, aspartate and glutamate metabolism	2(24)	0.0081	4.8	0.62879	0.17139	0.00285	Succinate; Fumarate

Propanoate metabolism	3(76)	0.0086	4.8	0.65986	0.17139	0.05093	Succinate; Lactate

Table S7. Age group II: significant metabolic pathways.

**Pathway Name**	**Match Status**	**p**	**-log(p)**	**Holm p**	**FDR**	**Impact**	**Metabolites**
Citrate cycle (TCA cycle)	3(30)	0.0000	11.5	7.7332	6.8391	0.19285	Pyruvate; Alpha-ketoglutarate; Fumarate
Alanine. Aspartate and glutamate metabolism	3(24)	0.0000	11.0	0.0013507	6.8391	0.00285	Pyruvate; Alpha-ketoglutarate; Fumarate
Butanoate metabolism	3(40)	0.0001	9.4	0.0064125	0.0021923	0.10287	Pyruvate; Alpha-ketoglutarate; Fumarate
Vitamin B6 metabolism	2(32)	0.0025	6.0	0.19135	0.049702	0.03828	Pyruvate; Alpha-ketoglutarate

Table S8. Age group III: significant metabolic pathways.

**Pathway Name**	**Match Status**	**p**	**-log(p)**	**Hol m p**	**FDR**	**Impact**	**Metabolites**
Glyoxylate and dicarboxylate metabolism	4(50)	0.0000	12.9	1.9207	1.9207	0.13906	Alpha-ketoglutarate; Glycolate; Oxalate; Citrate
Citrate cycle (TCA cycle)	2(20)	0.0010	6.9	0.076216	0.03859	0.14904	Alpha-ketoglutarate; Citrate
D-glutamine and D-glutamate metabolism	1(11)	0.0271	3.6	1.0	0.72364	0.0	Alpha-ketoglutarate

Table S9. Age group IV: significant metabolic pathways.

**Pathway Name**	**Match Status**	**p**	**-log(p)**	**Holm p**	**FDR**	**Impact**	**Metabolites**
Citrate cycle (TCA cycle)	2(20)	0.0004	7.8	0.031182	0.014345	0.17601	Alpha-ketoglutarate; Pyruvate
Alanine, aspartate and glutamate metabolism	2(24)	0.0006	7.5	0.044631	0.014345	0.0	Alpha-ketoglutarate; Pyruvate
Vitamin B6 metabolism	2(32)	0.0010	6.9	0.076816	0.016172	0.03828	Alpha-ketoglutarate; Pyruvate
Butanoate metabolism	2(40)	0.0016	6.4	0.11868	0.021098	0.08516	Alpha-ketoglutarate; Pyruvate
